# Cases at the Medical Dispensary, Medical Department of the University of Buffalo—Session 1848

**Published:** 1848-07

**Authors:** 


ART. III.—*Cases at the Medical Dispensary, Medical Department of the University of Buffalo—Session* 1848. *By the Editor.*

During the lecture session, at the Medical College of Buffalo, a dispensary for medical cases is open for indigent patients who may choose to avail themselves of it upon the condition of appearing before the class. Patients presenting themselves at this dispensary are examined and prescribed for by the Prof. of the Principles and Practice of Medicine; and on stated days, when they appear before the class, the cases are made subjects for practical instruction in diagnosis, pathology, and therapeutics. A considerable proportion of the cases, as would be expected, are sufficiently common, and, although not less instructive to the class on that account, would possess no novelty for the medical reader. Some of the cases, however, as would also be expected, were not without features suggesting practical considerations of interest to the practitioner, as well as the novitate. We purpose to select a few of the latter for brief notice and comment in the present article. Notes of all the cases presenting at the dispensary are preserved in a register devoted to that object, together with memoranda of the points to which the attention of the class was particularly directed. We select the cases from the collection as thus preserved, offering such remarks as suggest themselves in making this report. The cases are selected in order, as contained in the register, without following any arrangement.

*Case* 1. *Chronic Eczema.*—S. H. aged 14: Has suffered from cutaneous eruption for thirteen years. At first it appeared on the face and scalp; afterward on the upper and lower extremities simultaneously. For the three past years it has been confined to the lower extremities, mostly between the ankle and knee. States that his mother has always been subject to a similar affection, but less in degree. The eruption is manifestly Eczema. The affected parts are red, hot, and covered, in discrete patches, with thin incrustations. No elementary vesicles are discoverable.

He has had a great variety of treatment, but cannot specify what it has been. Has taken, among other things, thirteen bottles of sarsaparilla.

Directed (after remarks on the case) as follows: rigid cleanliness by daily ablutions of the affected parts; Fowler's solution gtt. v. three times daily; reduced citrine ointment.

This case, with the above history, presented itself at the session preceding the last, May, 1847. Under the foregoing treatment the patient rapidly improved, and reported well, June 24, 1847, for the first time for thirteen years.

In February, 1848, he again presented himself at the Dispensary. He had continued free from the disease up to the middle of January, when it again made its appearance in the same locality. Since then it has rapidly increased, and is nearly, if not quite as bad as when he first came under notice, in May 1847. He has also suffered, and still suffers from sub-acute laryngitis, being unable to speak above a whisper. He has no cough, no expectoration, no uneasiness in the larynx, nor embarrassment of respiration—nothing but the aphonia.

After some remarks on cutaneous diseases generally, and the diagnostic features of Eczema, it was concluded to prescribe the Liquor Potassæ Arsenitis, and to make no medicinal applications to the affected parts, but to enjoin rigid ablutions; the object being to test the therapeutic efficacy of the internal remedy in this case, conjoined simply with cleanliness.

March 4.—Limbs somewhat improved. A few days previous to this date, œdema of face occurring, the dose of Fowler's solution was diminished to three drops, three times daily. It was now decided to discontinue the internal remedy, and in order to see what effect will follow a local application exclusively pursued, the same remedy which was employed the previous spring was prescribed, as follows:—Ung. nit. Hydrarg, ʒ ij. ung. aquæ rosæ ℥ iss. M. Affected parts to be covered with oiled silk. Milk and farinaceous diet.

March 13.—Much improved. The local application has evidently been useful. Prescribed on this date, with reference to the Laryngitis, as well as the Eczema, the following:—Iodid. Potassii ʒ ss. Aquæ ℥ iv. Teaspoonful three times daily.

March 18.—Limbs nearly well. The cutaneous affection and the Laryngitis were both cured under the above treatment.

Remarks.—*A sine qua non* in the treatment of chronic Eczema, (and the same remark applies to Impetigo,) consists in the daily removal of the morbid exudations, by means of ablutions with simple water, or water rendered alkaline by potash or soda. The exudations should be dissolved and washed away, not removed by force, and care not to irritate by friction in the process is important. When the incrustations are thick and adherent, a poultice applied for several hours, or the warm water dressing, should precede the ablutions. Simple cleanliness, perseveringly continued, conjoined with regulated diet and regimen, will often suffice to effect a cure; without attention to these points, remedies, in a large proportion of cases, will be useless. One of the greatest obstacles in the treatment of obstinate cases of these affections, is the difficulty of securing cleanliness. Patients or friends will not take the necessary pains, and will insist that the cure shall be accomplished solely by medicinal appliances.

The local application which we have found oftenest useful in the two affections named, is the citrine ointment. It should be largely reduced Simple cerate may be employed to dilute it; but this preparation, as kept by apothecaries, frequently becomes rancid, or is made with rancid oil, and proves irritating to an excoriated surface. Hence, we are in the habit of prescribing the Ung. Aquæ Rosæ as the vehicle for the mercurial.

A nutritious, non-stimulating diet, is important, and living in the open air is generally an useful regimenal point.

Internal remedies may not be called for. The Liquor Potassæ Arsenitis frequently exerts in these, as in other chronic cutaneous affections, a surprising efficacy. The above case is interesting chiefly from its long duration, having existed almost from birth, and the promptness with which it yielded to the therapeutical course pursued. The patient presented no evidence of scrofulosis beyond the eruption, and his general aspect was not unhealthy.

*Case 2. Suspected Tuberculosis of Lungs in stage of incipiency.*—M. S., aged 18: Mother living and in good health. A brother died with consumption. Father died with the same disease at the age of 44.

Patient was never ill with any notable ailment, until present affection. Began to cough in June 1847. Cough has continued up to the present time, February 1847. The cough was for a long time dry; has expectorated for the last three weeks, most freely in the morning; character of expectoration not recorded. Has been more ill for the last three weeks than previously, in several particulars. Has felt debilitated, and general malaise. Chills have occurred on going to bed, followed by febrile movement, and sweating. These have occurred about every alternate night. Had Int. fever about four years ago, which continued three weeks. Has had no pleuritic stitches. Has lost flesh, but is not emaciated. Still continues at work. Appetite good. Bowels regular. Physical signs, at a single examination, negative.

The patient was a poor boy, and has been, heretofore, accustomed to peddle nuts, etc. for a livelihood. Of late he had been employed in a beer and cider establishment, the proprietor of which was willing to provide what might be necessary for the restoration of his health.

Remarks.—The question of prime interest and importance in this case, related to the diagnosis. Was the disease Chronic Bronchitis, or incipient Tuberculosis? The long continued dry cough, not succeeding an attack of acute bronchitis; the presumed herereditary predisposition; and the paroxysms resembling hectic, were circumstances exciting strong suspicion of the latter. The account is deficient as respects the state of the pulse, skin, &c. They probably presented no morbid symptoms, appreciable at the examination. Physical exploration, at a single examination, revealed nothing positive. The terberculous deposit could not, therefore, have been extensive; but it is well known that small tubercles may exist in large numbers, before coalescence, separated from each other by healthy lung, without being readily detected by physical signs. Repeated examinations, under such circumstances, frequently enable us to discover some deviation from the normal signs, which if constant, and fixed at a particular location, especially near the summit of the chest, although trifling in amount, authorize, when taken in connection with the symptoms, a positive diagnosis. A deduction of this sort, however, cannot be made from a single exploration.

Excluding physical signs, then, in this case, it was set down as a case of suspected incipient tuberculosis; but the diagnostic evidence not being conclusive, it was deemed advisable to prescribe as if it were simply a case of bronchitis.

He was directed to put on a flannel waistcoat, having worn nothing of the kind through the winter, and to take the following:—℞ Bals. Copaivæ ℥ i., Gum Arabic ʒ ij., S. Morphiæ gr. i., Spts. Lav. Comp. ℥ ss., Aquæ ℥ iii. ss. M. Teaspoonful three times daily.

Under this treatment, he rapidly improved. The febrile paroxysms shortly disappeared, the cough ceased, he recovered his flesh, and, March 28th, he reported well.

Judging this case by the result of the treatment, it was a case of sub-acute bronchitis, induced and kept up by exposure, with insufficient protection of the cutaneous surface. That it would have eventually terminated in pulmo-tuberculosis, is highly probable. Many cases of the latter disease among the poorer classes, are developed in that way.

In treating cases of Bronchitis, acute or sub-acute, the functions of the skin require especial attention. It is to be recollected that the skin and pulmonary mucous membrane are both excretory tissues, sustaining toward each other an antagonistical relation. By securing the active performance of the cutaneous functions in bronchitis, we not only do good by way of revulsion, but the inflamed tissue is relieved of its physiological duties by the increased and supplementary action of the skin. In acute bronchitis, the patient instinctively desires to keep the skin in a perspirable state, from the relief which he experiences. In sub-acute bronchitis, to preserve the temperature and vital activity of the surface is an important part of the treatment.

The balsamic remedies, when not contra-indicated by febrile movement, or irritability of the stomach, will often be found to exert an efficacy in chronic affections of the pulmonary mucous membrane, which may almost be styled specific.

*Case* 3. *Erythema.*—Mr. A., about 60 years of age. The disease commenced on one of the lower extremities, in April, 1848. It began with a small spot between the ankle and knee, somewhat reddened, epidermic secretion increased and modified, so as to be covered with dry scales. Now, on the extremity first affected, there is a space irregularly shaped, several inches in diameter, presenting a glossy redness, with a few, scaly, loose patches of epidermis. The redness momentarily disappears on pressure. It is attended with burning pruritus. On the other extremity, in the same locality, a small spot exists, precisely as the affection first commenced on the limb first affected—a spot somewhat reddened, covered with dry scales. He has taken large quantities of Sarsaparilla, Patterson's pills, etc. With a view to testing the efficacy of Fowler's solution in this case, it was prescribed in dose of 6 drops 3 times daily, and no medicinal applications to the part ordered. The above is the record made Feb'y. 26, 1848.

March 8th, No material alteration in the appearances of the affected parts. The following was prescribed as a substitute for the Fowler's solution:—℞. Iodid, Potassii ʒi, Decoct, Sarsae Comp. Oi. Tablespoonful three times daily.

To relieve the pruritus the following local application was directed:—℞. ung. aquæ Rosae ℥i, Kreosote gtt. xxx, M. To be applied after ablutions with warm water, or exposure to the vapor of warm water.

March 18th, Limb improved—Complains still of pruritus—Treatment continued.

March 25, Appearance of limb not so good as a week ago—More redness and pruritus—Disease shows a disposition to extend itself—Substituted for the treatment last pursued, as follows:—S. Quiniæ ʒi, Elix. Vitriol, fʒi, Aquæ ℥iv. Teaspoonful three times daily. For a local application, the following:—℞. Ung. citrine ʒi, ung aquæ Rosæ ℥i, Kreosote gtt. xxx. Parts to be covered with oiled silk.

April 1, Somewhat improved—Continue treatment.

April 8, More improved—Directed as follows:—℞. Sulph. Quiniæ ʒ ss. Citratis Ferri ʒ i. Aquæ ℥ iv. Teaspoonful three times daily—Local application continued.

April 16, Much improved—Treatment continued.

This patient subsequently was discharged quite well.

*Remarks.*—The result of the treatment goes to sustain the opinion of the dependence, in this instance, of the affection upon general debility as its constitutional cause. As a general remark with reference to the management of cutaneous affections (and the remark is by no means limited in its application to cutaneous affections) that treatment will be most successful, or alone successful, which is adopted with reference to the constitutional aberration of which the disease is merely an effect, or local expression.

*Case* 4. *Pulmonary Emphysema, Asthma.*—R. A. S., aged 44, Joiner. Has suffered from dyspnœa ever since his earliest reccollections. It is apt to occur after exercise, not in severe paroxysms, but much worse at some times than at others. Always greatly increased when the atmosphere is humid, and when he contracts a cold. Has always had cough and expectoration. When he does not expectorate, suffers much more from dyspnœa. Has always kept at work. Has been treated formerly by Thomsonian practitioners, Never has consulted a regular physician until now, but, since losing confidence in Thomsonism, .has resorted to a variety of patented nostrums. Is not much emaciated. Muscular strength pretty good. Always suffers most at night from dyspnœa. Is obliged to lie with his head elevated, and sometimes to sit up all night. Chest presents good resonance, marked general amplitude, with the sibilant and sonorous rales. Sounds of heart normal. This was the record March 22. The diagnosis was Emphysema. After remarks on the various morbid conditions with which Astmatic affections are connected, the patient was advised to try the inhalation of Chloroform moderately, to relieve the dyspnœa.

On the 23d. he reported that he found considerable relief from the chloroform. The copaiva mixture was then prescribed; the inhalation of chloroform to be continued.

March 25, Reported much improved. Is able to sleep comfortably at night, and thinks he is able to go to work. Has used the chloroform frequently since last report.

Remarks.—This patient did not again present himself. The case offers nothing peculiar, nor interesting, except the use of the chloroform as a palliative. In so far as this single instance affords any grounds for a deduction, it would seem that this agent is well adapted to that object. The philosophy of its applicability would seem to involve spasm, as an element of astmatic dyspnœa, dependent upon muscularity of the smaller bronchi. The latter, although still mooted, we suppose has been abundantly demonstrated.

*Case* 5, *Azoturia.*—The subject of the following case did not present himself at the Dispensary. He was a patient of Dr. Sprague, of this city, to whom we were indebted for specimens of urine, which were exhibited to the class, illustrating a rare and interesting variety of urinary disorder. The first specimen was received on the 25th February. Its sp. gr., as determined by the urinometer, was, 1,030. It deposited no sediment, resembled porter in color, and emitted a strong urinous odor. On placing a portion in a watch glass, and adding an equal quantity of nitric acid, two thirds of the mass became solidified in about half an hour. The solid deposit presented an appearance of minute crystals, (nitrate of urea). The test was repeated several times with the same result.

March 1st. Another specimen was obtained, which contained a mucus deposit, at first white, but which afterward became red. On shaking the vial the sediment disappeared, and the urine became somewhat turbid. Sp. gr. 1,030. On testing as before, the same results were obtained.

Dr. S. furnished verbally the following account of the patient:—He is a German laborer, and had suffered for some time from indefinite ailments, such as indigestion, palpitations, neuralgic pains in chest, etc. He has constantly wasted in flesh. He had been a patient of a Dutch uriscopic empiric, who had examined the urine only as regards its sensible appearances. Dr. S. was treating him with tonics and anodynes, under which treatment his health was manifestly improving.

REMARKS.—This is a very rare form of disease. Dr. Prout, in his immense experience with urinary affections, met with it in but a few instances. He states that he has met with Diabetes Mellitus twenty times to this disease once, and Diabetes is a very rare disease. Dr. Prout thinks it precedes Diabetes in some cases.

The affection involves mal-assimilation, or disorder of the molecular changes incident to nutrition. Adopting Dr. Prout's nomenclature, it denotes defect in either the "primary" or "secondary assimilatory processes," of which the increased quantity of urea in the urine is only a consequence. Emaciation must necessarily ensue as a result of its continuance.

It may be supposed that the neuralgic pains, the palpitations, etc., are due to the urea, circulating with the blood, acting on the nervous system. We regret that we are unable to give a history of the case with more detail. The treatment reccommended by Dr, Prout is (first) to avoid rough treatment: (second) tonics and anodynes. The latter had been employed in this case with good effect.

The case exemplifies the importance of examining the urine in cases of indefinite disease accompanied with wasting, with a view to this disorder.

*Case 6, Tuberculosis of Lungs.*—We select the following from the cases of tuberculous disease of lungs, as furnishing some points of interest.

G. W. C. aged 25. The general history was as follows;—When seventeen years of age he began to experience pain in the right side of chest. Before this, his health, from childhood, had been perfectly good. The pain was sharp, nearly constant, but not so severe as to lead him to apply for medical aid for eighteen months. He then placed himself under the care of a physician, who vesicated the chest, and afterward prescribed strengthning plasters. He took no internal remedies. The pain was relieved by these measures, and for some years afterward he felt no pain, but was conscious of a weakness in the part.

In 1844–5, had recurrence of the pain during the winter, affecting both sides of the chest, which continued four or five months, and passed away in warm weather. Took only tar water, and a patented preparation of tar and liverwort.

Went to Mexico, with the army, in the autumn of 1845, as clerk in quartermaster's department. He was subject to great exposure in Mexico. His habits of life there, and previously, were very irregular. During the winter of 1845, had recurrence of pain in the chest, with cough, and expectoration of matter streaked with blood. Had had some cough and expectoration from the commencement of the disease, varying much in amount at different times. In the spring of 1846, he had, what was called rheumatism of hip and knee joints, affecting these joints only. During summer of 1846, he was free from pain in chest, but was lame all summer from affection of hip and knee joints.

In the winter of 1846–7, he lived at Cincinnati. Suffered from pain in chest, at times, but not severely.

In the summer of 1847, felt nothing of the pain in chest; but on coming to this city, in the autumn of the same year, he began almost immediately to experience pain in chest, which was quite severe. He had, at the same time, more cough and expectoration than usual. More or less cough had existed all along; more at some times than at others, and at times nearly, if not quite absent. Pain has frequently shifted its location, and has always been sharp in character, whether severe or not.

In January, 1848, he was seized suddenly in the street with hæmoptysis. Expectorated blood copiously. Slight attacks of hæmorrage occured for three or four days subsequent to the first attack. At this period, he consulted Dr. Lockwood, of this city, by whom he was advised to apply at the dispensary for examination, in April, 1848. In the meantime, since January, he had had two attacks of hæmoptysis, one about two weeks after the first attack, the other about two weeks since. The expectoration for the past month has been more copious than before. Its characters are not recorded. Has pain in chest still at times. It occurs, whenever he makes muscular exertions. Appetite is tolerable. No evidences of indigestion. Bowels regular. Tongue furred.

Pulse 100, small and feeble. Had not observed any febrile movement at night. Has perspired for three or four nights past—not before.

His respiration is hurried on exertion. Is easily put out of breath.

Stated that he had lost a good deal of flesh—he estimated 30 pounds—

chiefly the last winter. Does not present the appearance of marked emaciation.

Has never had chills. His father not living—died from an accident. Mother still living, and in good health. Family not prone to pulmonary complaints. A brother expectorated blood many years ago, but recovered, and is still in good health.

On physical examination the resonance seemed duller than would be expected, in health, on both sides anteriorly, but slightly duller on the right, than on the left side. Sounds of the heart very audible on the right side. Respiration on the right side extremely feeble, and, for the most part, inappreciable. Vocal fremitus particularly perceptible through the stethoscope, on the right side. On the left side respiration feeble. Expiratory murmur not perceptible. Posteriorly slight sub-crepitant rale on the left side. Respiratory murmur feeble on both sides, and expiratory murmur not perceptible.

Remarks.—The diagnosis in this case was tuberculosis, not associated with a strong tuberculous diathesis. The history does not exhibit the usual course of progressive phthisis. The disease has been induced, in a great measure, probably, by exposure, and irregular habits of life. It is a case in which the physical signs, although not of a striking character, are yet quite decisive; and it is by means of these signs, chiefly, that the diagnosis is rendered positive. The tuberculous deposit, although existing in both sides, is larger in the right side.

The case illustrates the determinate value which attaches to certain physical signs, small in amount, when grouped together, and connected with rational symptoms. The slightly relative dullness on the right side, the feeble respiration, vocal fremitus, propagated sounds of heart, suffice to render the diagnosis positive, even upon a single examination, especially when taken in connection with the history.

The pains in the chest, which appear to have preceded, and which accompanied the tuberculous disease, is a singular feature of the case. It would hardly seem that they could be wholly due to pleuritis supervening upon the tuberculous deposit, but were probably of a neuralgic character.

As the history of the case does not disclose a strong constitutional tendency to phthisis, it is possible that the progress of the disease may be retarded so that life may be prolonged for an indefinite period. The non-use of medicinal agents except those of a tonic nature, regulated habits of life, nutritious diet, and living in the open air, with the observance of hygienic measures of every description, in our experience, furnish the most reliable means for effecting this result, which, although by no means frequent, still occurs sufficiently often to warrant, in many cases, sanguine hopes of its attainment.

The patient did not present himself except on the occasion when the foregoing examination was made, April, 1848.*

*Case 7. Prurigo Formicans et Senilis.*—The following case is only interesting as respects its history and diagnosis. The remedies which had been employed previously to the application of the patient at the dispensary, were not known, nor has the result of those suggested at that time been communicated to us. We select the case on account of its presenting a rare form of cutaneous disease—rare, at all events, in our experience.

A. W. H. Farmer, aged 54. Enjoyed pretty good health until five years ago, when he began to suffer from his present disease, Thinks his constitution has been somewhat injured by hard labor.

The eruption first appeared on the face, and more particularly about the eyebrows. For sometime before the first appearance of the eruption, and afterward, he suffered from depression of spirits, dullness of mind, disposition to sleep, etc.

He has suffered from the disease, for five years, during the whole of the winter season, and more or less through the summer. The periods of intermission in the summer season, have been annually less and less. Until the last winter, the eruption had been limited to the face. For the last winter it has affected the arms, especially the forearms, and also the inner side of the thigh. Appetite and digestion all the time good, and bowels regular. Appearance of the urine natural.

The pruritus is intense, especially at night; so much so that sleep has been almost impossible for weeks in succession. He cannot sleep in a room heated by a close stove, nor can he sit in one during the day without intense suffering from pruritus. The pruritus is accompanied by a sensation as if small insects were creeping under the skin: this is especially the case on the face. Skin becomes at times thickened and chapped.

The characters of the eruption are as follows:—Small, hard, round papules, of the color of the skin. No papules at the examination were perceptible by the eye, but the skin feels roughened on passing the hand over it. Small dark scabs are thickly scattered over the face, and forearms, presenting an appearance highly characteristic.

The patient has abstained from animal food for the last three years. He has found by experience that he was worse when he ate meat. He has

*We have lately been informed by Dr. Lockwood that this patient is declining, being now unable to leave his bed.

taken a great variety of medicines, and employed all sorts of practitioners. Has applied an infinite number of washes. Does not know the nature of the remedies he has taken, nor the composition of the washes. He has received little or no benefit from anything he had done.

The patient resided in an adjoining county, and the case was not afterward heard from. The treatment recommended, was a faithful trial of the Liquor Potassæ arsenitis. If this fail, the carburrett of Potash, Iodide of Potassum, and the mineral acids were suggested. The warm bath perseveringly employed, was also advised, and acetic acid diluted, as a wash to relieve the pruritus.

Among the different species of the genus prurigo are two, called *P. sesilis* and *P. formicans*. The former term is applied to the disease as it presents itself in the aged. This species is peculiarly obstinate, and often incurable. The latter term denotes a peculiar pruritus which is compared to the crawling of insects under the skin. The patient in the foregoing case described such a sensation, and as he was somewhat advanced in years, the disease may be said to be of both species.

*Case* 8. *Diabetes Mellitus.*—B——, a German, aged about 30, a joiner, unmarried, presented himself, April 6th, with the above disease. The urine was tested by yeast, and exhibited evidence of the presence of sugar in abundance. The quantity of urine was greatly increased, but the precise quantity expelled in a given period, was not ascertained. Patient stated that he thought he passed about four quarts during the night. The urine was frothy on agitation, and exhaled the characteristic fragrant odor, something like newly mown hay. The patient's breath had the same odor. Sp. gr. was obtained, but not recorded.

The disease had been known to have existed for seven months. It was preceded, as he stated, by an attack of pain in the chest and fever—inflammation of the lungs, as he was told by his physician. Six weeks after this attack, he first noticed that he voided a larger quantity of urine than usual. Had never observed any sediment. His health had been good up to the febrile attack just mentioned. Had lost some flesh, not much; precise quantity lost not known. His present symptom, were as follows:—Appetite pretty good: Suffers from indigestion and regurgitation of food: Complained of a bad taste in mouth, which he could not describe, said it resembled, somewhat, that of a rotten egg: Burning pain at epigastrium: Gums swelled, red and spongy: Suffered from constant thirst: Bowels very costive: Fœcal matter dry and hard. Restless and irritable: spirits depressed: sleeps sometimes well, and sometimes badly: slight cough, which is dry: catarrhal discharge from nostrils: skin extremely dry and rough: pulse accelerated, and somewhat vibratory.

He had not, as yet, abstained from diet containing saccharine principles. The treatment recommended was as follows:—Animal food, eggs, rice and hard biscuit, for diet; the warm bath daily, and the citrate of iron in combination with sulph. quinine.

One month afterward, the patient again presented himself, stating that he had faithfully followed the above course of treatment; that he had gained in strength, but not in weight; the quantity of urine had diminished about one half. It was not tested at that time, but its appearance, and peculiar fragrant odour, demonstrated the presence of sugar. The odour of his breath was still noticeable, and was so obvious, that he was recognized by this circumstance, before we could call to mind the case from recollection of his countenance. His teeth had that whiteness which belongs to this disease. He applied at this time to know if a sea voyage would be likely to be serviceable, and, if so, he purposed returning to Germany. He was encouraged in this idea, and left for the seaboard.

Remarks.—The presence of sugar in other secretions than the urinary secretion, and in the blood, which late researches have abundantly demonstrated, render it inappropriate longer to rank Diabetes among Renal diseases. It is a disease affecting the assimilatory processes, in consequence of which sugar ingested passes unchanged into the bloodvessels, and alimentary principles capable of being converted into sugar undergo that chemical change. The kidneys are involved only as one of the excretory outlets by which this useless saccharine material is eliminated from the system. The hyper-secretory action of the kidneys, (which is probably due to the presence of the sugar acting as an excitant,) constitutes an important element of the disease. This will serve to explain, in part, the dryness of the surface, the costiveness, the thirst, etc. The loss of those alimentary principles which contain sugar, or are capable of conversion into sugar, for all purposes of healthy assimilation, will explain the debility, the wasting, and the great gravity of the disease. We have thus advanced a considerable way in our knowledge of this disease; but we are still at a distance from knowledge of the nature of the perversion which the processes of assimilation undergo, and of the ulterior morbid condition upon which this perversion depends. In other words, its true pathology, and, as a matter of course, its ætiology are still unknown. The knowledge, however, which has been acquired is not without practical value. It prevents, in the first place, treatment based upon the idea of its being essentially an urinary disorder, and other false pathological views, and in this way saves the patient from measures which might prove not only useless, but injurious. This, although negative, is, nevertheless, not the least important of the advantages which attend improved knowledge of diseases generally. But it leads to a positive advantage in teaching the propriety of withholding, in as far as practicable, aliment of a saccharine nature, and aliment capable of being readily converted into sugar. In this way the quantity of sugar in the urine is almost sure to be considerably diminished, and, as a consequence, the amount of urine is lessened. Although the measure is but palliative, it is vastly important, inasmuch as, in itself, it serves to prolong the life of the patient, and gives time for the employment of means by which, it may be hoped, a cure will be effected. With reference to the latter, science gives but little precise information, and experience not much encouragement. Our rational course, in the present state of knowledge, is to endeavor to restore the proper action of all the functions, and, especially, to modify and improve the processes of assimilation. For this end, tonic remedies, nutritious diet, and hygienic regulations of every description, are essential.

*Case* 9. *Spinal Affection.*—The following case is given as illustrative of a class of cases, sufficiently numerous to be frequently met with by every practitioner, for which it is difficult to find, nosologically speaking, a local habitation and a name. They have been called at different times by different appellatives, and are distinguished among different practitioners by different titles. Formerly, it was fashionable to include them under the term, dyspepsia. Of late, they have been oftener classed under the head of spinal irritation. We have adopted of late years, the latter plan, using the expression *spinal affection*, as less involving hypothesis concerning the pathology. The pathological relations of the spinal cord are involved; but we are not prepared to say that the affection is primitively located in the spine. The cases referred to are distinguished, at first view, rather by the great diversity of symptoms which are presented, than by the close analogy which they have with each other. The phenomena are infinitely varied. They are united, however, by some points which are common to all. Some of the most important of these are as follows:—The phenomena involve, par excellence, the nervous system, and are constantly varying in the same case, being widely different at different times. They are attended with a peculiar sense of debility, consisting of a want of the consciousness of ability to exert muscular power, rather than an actual dimunition of that power; and this sense of weakness comes and goes, being present on some days, and not on others, and at some portions of the same day, and not at other portions. The symptom alluded to, is, perhaps, located in the mind, rather than in the muscular system, and an inability to exert the mental powers is equally characteristic. Depression of spirits is almost invariably present. There is almost uniformly tenderness on pressure over some portion of the spine, which, however, in its degree, bears no constancy of relation to the other symptoms. These are some of the positive symptoms which are common to all cases. But the cases are associated together, negatively, by the absence of evidence of inflammation, or lesions of the organs whose functions are disturbed, and by the fact that the function of nutrition is seldom impaired by the affection, even when protracted. British writers describe the malady under the title of Hysteria, or the Protean disease. In some of its forms it may be embraced under the head of Hypochondriasis. By means of the physiological and pathological relations of the spinal cord, we can explain many of its phenomena; but we suppose the cord to be generally affected secondarily, and the most frequent general condition which obtains is anæmia. But we cannot undertake to write a dissertation on the affection in this connection, and will now proceed to the case, to which we will append a remark or two, if space permits.

S. aged 40, shoemaker. "Never knew a day's illness up to six years ago last April." He was seized suddenly with chills, and pains in epigastrium and left side. The chills continued only a short time, but the pains continued through the night, recurring in paroxysms with a few moments intermission. He was attended at that time by Dr. A. He was confined to his house a month by weakness, and then came out to his shop, but felt too weak to walk home, and was carried back. After that he was confined to his bed all summer, with, as he says, weakness. Had no pain, and appetite pretty good. In August he first began to walk about the room. Was lifted out of bed, and carried out of doors in a chair frequently during the summer. Dr. L. attended during this summer.

In the winter he was able to walk about town. Dr. W. attended during this winter. The next summer he walked about, except at times when he was unable to do so, as he states, from debility and "pains all over." Had no medical attendance during that summer. This was in 1843. In the winter of 1843–4 he was attended for several months by a "Botanic Practitioner." Took one steam bath, and and various remedies. During the latter of winter and in the spring of 1844, had no medical attendance. Says he was able to get about a part of the time, and a part of the time he was unable, from debility and universal pains. In the summer of 1844, he improved, but suffered in damp weather invariably. Had no medical attendance.

Went to England in October, 1844. In the winter of 1844–5, was under the care of Dr. Horner, of Hull, for 8 months. Health about the same as during previous winter. Sometimes could walk about pretty well, at other times states that he would be suddenly arrested when walking, and be unable to move for several minutes: seemed to lose voluntary power over the muscles, and the effort to exert it produced pain. Took considerable medicine, but does not know what it was. States that when warm he could use his limbs freely, but when in the cold air, to quote his language, "his joints and cords were fast."

In summer of 1845, went to Lowth, into dispensary under charge of Dr. Banks, and continued under his care six or eight weeks. Dr. B. advised him to go to Woodhall spa, in Lincolnshire. Suffered through the summer much of the time from weakness and pain. Remained at the spa two weeks. Went in the autumn of 1845 to the Horncastle Dispensary, under charge of Dr. Barton. Continued until July, 1846. Could get about but little through the winter, but was better during the summer of 1846. In August returned to this country. In winter of 1846–7, sometimes could get about, and sometimes not, from debility and pains. Dr. A. advised in February, 1847. In summer of 1847 could walk about part of the time, but felt unable to do any labor. No medical attendance.

Winter of 1847–8. Had been under the care of Mr. Weyburn, (who treats rheumatic affections by vapor baths and shampooing) from November 10th up to May 10th, the time of his application at the College Dispensary. Had been during the winter about the same as during previous winters since the time his morbid history commences. His present symptoms, as recorded May 10th, were as follows:—Aspect rather pallid. Thought he weighed about 20 pounds less than when in health, but did not know his exact weight in health. Complexion not unhealthy. Appetite good. Food occasionally causes oppression. Bowels now costive, but can hardly tell what their natural condition would be, as he has been constantly taking cathartic medicine through the winter and spring. Tongue slightly furred.

Suffers occasionally from slight headach. Sleeps generally pretty well, but sometimes troubled with vigilance. Suffers from pains in chest on each side. The surface of the chest is tender on pressure, pains unaffected by respiration. These pains have been pretty constant since December last; at times they are severe, preventing sleep. Sometimes they extend to epigastrium. Pulse 70, nothing abnormal. Sounds of heart, and impulsion, normal. Cannot learn that he has ever had febrile movement. Has occasionally been troubled with palpitations.

Has no cough. Occasionally, after eating, has experienced some slight embarrasment in breathing. Skin presents nothing unusual. His urine during the winter deposited a sediment. He brought a specimen at the time, and it proved to be the Lithate of Ammonia.

There is considerable tenderness on pressure over the upper portion of the dorsal spine.

The treatment directed, was S. Quiniæ and Citrate of Iron, and the application, first of Ung. tart. Ant. over the tender portion of the spinal column, and afterward of Croton oil. The Citrate of Iron and Quinia was subsequently displaced for the sulphate of Iron. Under this treatment, he reported amelioration of the pains in the chest. He was able to be about, but made little or no progress in advancing towards a condition to consider himself able to do anything for his own support

Remarks.—This case supplies a theme for extended remark; but we have already occupied so many pages with this article, that we must be very brief.

It illustrates a state of mind, which may eventuate in Hypochondriasis, but which, in its lighter shades, is not so well appreciated. A portion of mankind are able to preserve their energy and activity, notwithstanding disease —they struggle with it, they resist it, they fulfil their duties in spite of it, until compelled to yield, as all must do, sooner or later. Another portion of mankind, present quite a contrast to the foregoing. With them, disease, or even disorder is an insurmountable obstacle. They have no energy to struggle manfully against its influences; they seem to cherish rather than oppose it, and give way to a distrust of their ability to accomplish anything by the force of the will. Life, which is at the best, but a leasehold, is held by a large proportion of mankind under a heavy encumbrance; yet, notwithstanding this, some make it a profitable and even agreeable investment, while others are discouraged and paralyzed by the idea that their bodies lie under a mortgage to disease.

That some forms of disorder tend, more than other forms, to produce a state of mind unfavorable to energetic action and happiness, is true, but it is also true that differences in the mind itself, impose differences in the mental, and even physical characters of disease in different individuals.

Not to amplify upon an abstract view of this point, we have, if we do not err, in the foregoing case, an instance illustrating a want of mental stamina sufficient to triumph over morbid sensations. The patient, for six years, has felt disqualified for any exertion. His complaints are not imaginary, but on surveying the symptomatology of the case, nothing appears which necessarily compromises, to say the least, a limited activity. A person differently constituted would make the most of what physical powers he still possesses; but this patient, physically speaking, must be *aut Cæsar aut nullus*—he thinks he can do nothing until he recovers perfect health. He therefore folds his hands, and sits brooding upon his ailments, passively waiting for health. Under such circumstances, health will probably be long in arriving.

Patients of this description usually consult many medical practitioners in succession. Each new adviser commences with hopes of speedy success; and for a time, novelty seems to have a good effect; but after a while, it is very apt to be the case that patient and physician become mutually tired of each other, and a change is agreeable to both parties. With different practitioners, the affection receives different names. With one it is *liver complaint;* with another, *dyspepsia;* with another, *debility;* with another, *hypocondriasis;* etc. Treatment based upon the views which these names express, is often not nugatory, but hurtful. On the other hand, treatment based upon recognition of the mental characters of the affection, and an appreciation of the pathological relations of the spinal cord, will, to say the least, not be likely to expose the patient to injury from medicinal agents.

Patients of this class not unfrequently go the rounds of empiricism, patronizing in succession, the Steam doctor, the Botanic, the Uriscopist, and the Homœopathist; and it is this class which contribute not a small share of the profits of the nostrum maker, and the nostrum vender.

With reference to the proper plan of treatment, we will only say, in brief, that it embraces the observance of Hygienic laws; tonic remedies, and especially chalybeates, (being so often associated with anæmia); counter-irritation over the spine; diversion of mind by occupation; removal of groundless apprehensions; encouragement, and the *development of more mental energy and strength of will.* That portion of the treatment addressed to the *morale*, and especially that which we have Italicised, expresses what is wanting for recovery, rather than what the physician can be expected to impart. If the view of the affection which we have hastily sketched be correct, it often involves a defect in mental character, for which our materia medica furnishes no specific remedies, and which will be likely to resist, in many cases, any moral influence which the physician is able to exert.

